# Reconstruction and inference of the *Lactococcus lactis* MG1363 gene co-expression network

**DOI:** 10.1371/journal.pone.0214868

**Published:** 2019-05-22

**Authors:** Jimmy Omony, Anne de Jong, Jan Kok, Sacha A. F. T. van Hijum

**Affiliations:** 1 Top Institute Food and Nutrition (TIFN), Wageningen, The Netherlands; 2 Department of Molecular Genetics, Groningen Biomolecular Sciences and Biotechnology Institute, University of Groningen, Groningen, The Netherlands; 3 NIZO, Ede, The Netherlands; Purdue University, UNITED STATES

## Abstract

Lactic acid bacteria are Gram-positive bacteria used throughout the world in many industrial applications for their acidification, flavor and texture formation attributes. One of the species, *Lactococcus lactis*, is employed for the production of fermented milk products like cheese, buttermilk and quark. It ferments lactose to lactic acid and, thus, helps improve the shelf life of the products. Many physiological and transcriptome studies have been performed in *L*. *lactis* in order to comprehend and improve its biotechnological assets. Using large amounts of transcriptome data to understand and predict the behavior of biological processes in bacterial or other cell types is a complex task. Gene networks enable predicting gene behavior and function in the context of transcriptionally linked processes. We reconstruct and present the gene co-expression network (GCN) for the most widely studied *L*. *lactis* strain, MG1363, using publicly available transcriptome data. Several methods exist to generate and judge the quality of GCNs. Different reconstruction methods lead to networks with varying structural properties, consequently altering gene clusters. We compared the structural properties of the MG1363 GCNs generated by five methods, namely Pearson correlation, Spearman correlation, GeneNet, Weighted Gene Co-expression Network Analysis (WGCNA), and Sparse PArtial Correlation Estimation (SPACE). Using SPACE, we generated an *L*. *lactis* MG1363 GCN and assessed its quality using modularity and structural and biological criteria. The *L*. *lactis* MG1363 GCN has structural properties similar to those of the gold-standard networks of *Escherichia coli* K-12 and *Bacillus subtilis* 168. We showcase that the network can be used to mine for genes with similar expression profiles that are also generally linked to the same biological process.

## Introduction

*Lactococcus lactis* MG1363 is a worldwide studied plasmid-free derivative of the dairy starter strain NCDO712 [1]. Several genomes of *L*. *lactis* strains, including MG1363, have been sequenced to completion [2–4] and many regulons of *L*. *lactis* MG1363 are well characterized [5,6]. Still, the functions of many genes in its genome remain poorly understood. Reliable prediction and assignment of gene function remains a challenge deeply rooted in computational biological methods such as gene annotation and comparative genomics. Another option for gene prediction and function assignment is to construct gene co-expression networks (GCNs) [7–9]. A GCN is a graphical structure consisting of genes (depicted as nodes) and co-expression relationships, depicted as edges. The most connected nodes are the hubs, which generally correspond to genes encoding transcription factors (TFs) that drive the expression of the genes to which they are connected. Co-expression networks are used to characterize gene neighborhood relationships (commonly referred to as guilt-by-association) [10], which can be used to identify genes/proteins with similar functions and/or physical interactions [11]. A biologically meaningful network should be highly structurally organized, with clusters of genes (or modules) and genes connecting those clusters [12–15].

For reconstructing a GCN, Pearson or Spearman correlation coefficients are the most widely used measures of association to quantify gene co-expression [16,17]. Reconstruction of co-expression networks involves determining associations between genes based on their expression profiles. Studies on uncovering directional regulatory effects often focus on small-sized networks (with less than 200 genes). Several methods exist to infer activation and repression mechanisms in networks, but this is not the focus of our work here [11,18]. Inter- and intra-modular connections within a network complicate determining module boundaries [19]. Inter-modular connections are edges that connect genes belonging to different modules and intra-connections refer to edges that link genes within the same module. The presence of more connections within, rather than between, modules enables reliable module detection due to increased modularity (*Q*) of a network [20]. In addition to the Pearson or Spearman correlation approaches to reconstruct co-expression networks, other popular methods are GeneNet [21], SPACE [22], WGCNA [23] and ARACNE [24]. The choice of which of these methods to use for network reconstruction can be influenced by various factors, such as data size or whether one needs to infer regulatory directions between genes. For instance, Allen et al. [25] found that, for small networks consisting of less than 100 genes, GeneNet and SPACE out-perform the WGCNA and ARACNE approaches. Each network reconstruction method has its strengths and weaknesses [26,27]. Bayesian Network-based approaches like BNArray [28], B-course [29], Bayesian Network Toolbox [30] and Werhli’s BN implementation [31] perform relatively poorly for large networks [25]. Data quality and dimension, network size and robustness of the used reconstruction method all affect the quality of the network [32], while lowly expressed genes are known to introduce bias and reduce network accuracy [33].

Here, we present the *L*. *lactis* MG1363 gene co-expression network based on data from the NCBI Gene Expression Omnibus (GEO) database [34] and discuss its structural properties in comparison to two gold-standard bacterial networks, namely those of *Bacillus subtilis* 168 and *Escherichia coli* K-12. We expect this *L*. *lactis* MG1363 GCN to provide an excellent basis for data mining and a template for designing further experiments. Such experiments would particularly be focused on sub-networks or on the functional analyses of specific genes of interest.

## Methods

### Transcriptome and regulon data sources

Transcriptome data used for the *L*. *lactis* MG1363 GCN reconstruction was obtained from the NCBI GEO database (http://www.ncbi.nlm.nih.gov/), Table A in S1 File. The GEO accession numbers of the 64 experiments used are given in the header of this table and give access to the detailed descriptions of the experiments. The raw data was Lowess-normalized and scaled as described in [35]. The resulting normalized signals were used for the network reconstruction. To ensure a fair comparison between samples, experiments were grouped by (i) the growth medium used (GM17 or G-CDM, a rich and a chemically defined medium, respectively, containing 0.5% glucose), and (ii) the growth phase from which the samples were taken, namely early-, mid-, late-exponential or stationary phase, or based on ranges in culture optical density (OD). The processed data encompassed 64 conditions, after computing the median expression values per replicate and excluding datasets with genes with noisy expression. Downstream analysis of the data was performed using T-REx [36].

### Biological network reconstruction

The *L*. *lactis* MG1363 networks were generated in R language v3.0.2. Network structural properties were analyzed using R’s igraph package (version 1.0.0) and visualized in Cytoscape v3.2.0 [37]. Network density, modularity, average path length, diameter and number of detected modules were calculated for five methods, namely Pearson correlation, Spearman correlation, GeneNet, SPACE and WGCNA. We compared the networks thus generated and ranked them for performance. Networks generated using the Pearson or Spearman correlation coefficients were assessed by comparing the results of the degree distribution of the networks resulting from these two approaches to those of the power-law distribution [38,39]. To generate networks with the other three methods, the association parameters were varied. Only networks generated using specific regions of threshold parameters were considered for further analyses; hence, GCNs with (i) very high connectivity, (ii) low modularity, and (iii) very sparse connectivity (only a few hundred genes) were discarded. To examine the structural robustness of the *L*. *lactis* MG1363 network, a probabilistic random edge addition was performed using the approach described in [40]. A topological overlap matrix showing the degree to which directly linked nodes are connected was created to perform this analysis. In the WGCNA approach, we used a soft threshold approach on the adjacency matrix [23], which is a derivative of the topological matrix. Since the performance and reliability of network module detection methods are known to vary [25,41], we used at least four approaches to partition the networks, namely Walk-trap [42], Fast-Greedy [43], Infomap community [44] and label propagation [45].

**Fig 1:**
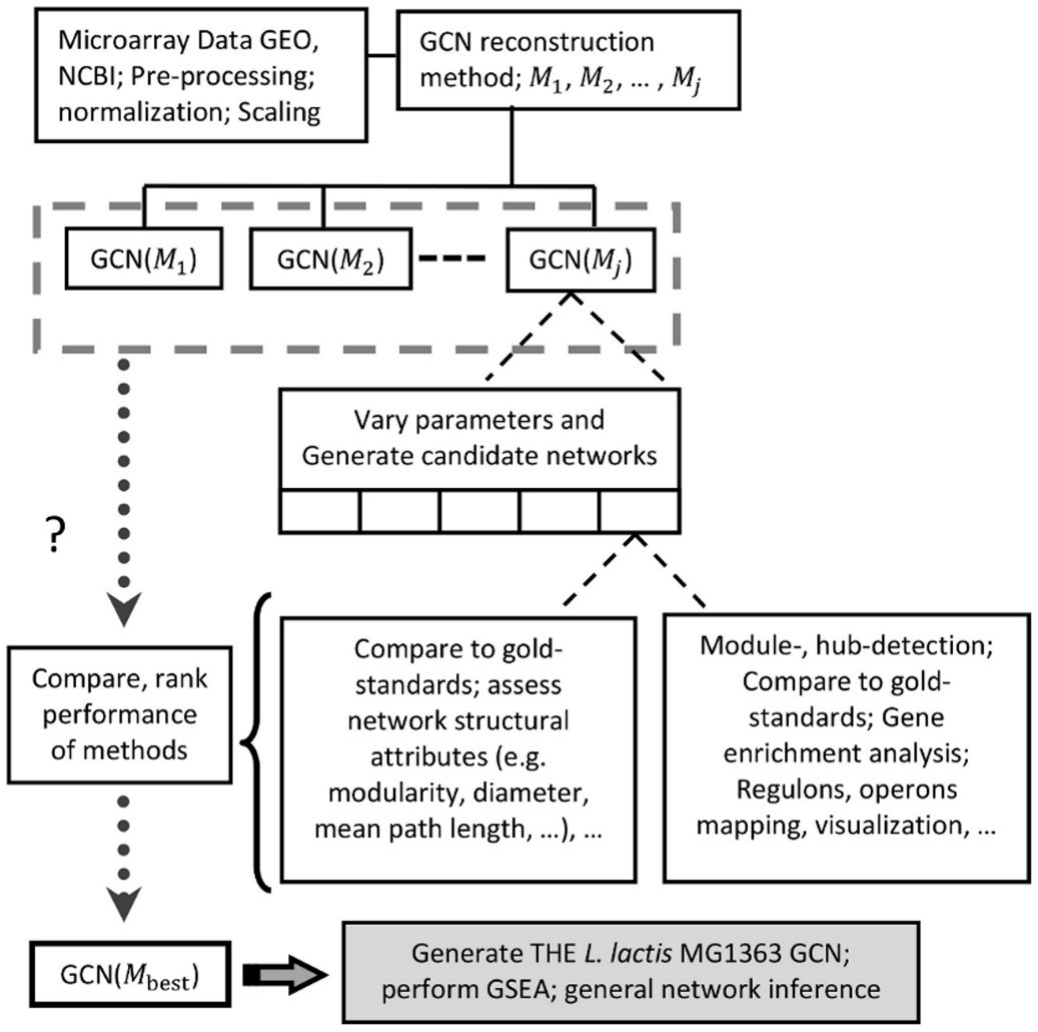
Workflow of gene co-expression network (GCN) reconstruction using different methods.

Data for regulatory network reconstruction of *E*. *coli* K-12 were obtained from regulonDB (http://regulondb.ccg.unam.mx/menu/download/datasets/index.jsp) [46], those for the reconstruction of the *B*. *subtilis* 168 regulatory network from the SubtiWiki database (http://subtiwiki.uni-goettingen.de/) [47]. Gene-set enrichment analysis (GSEA) was performed using the Genome2D web-server (http://genome2d.molgenrug.nl/). The summarized workflow is presented in Fig 1.

### Analysis of enriched network motifs

The detected network modules were subjected to DNA sequence motif enrichment analysis. We used MEME version 5.0.3 (http://meme-suite.org/) [48,49] for all network modules with at least four genes. Upstream regions of all genes within each module were extracted and used for the motif enrichment analysis (http://genome2d.molgenrug.nl/). The MEME search for motifs with a length between 6 and 16 bp was done on the upstream intergenic region, which are of variable length. Only the best motif of each cluster is reported—excluding the RBS motifs. Subsequently, the selected motifs we screened against the prokaryote TFBS database of PRODORIC Release 8.9 (using the TomTom Motif Comparison Tool (Version 5.0.4) of the MEME suite with default setting).

## Results and discussion

### Construction of *L*. *lactis* MG1363 gene co-expression networks (GCNs)

Publicly available DNA microarray data on *L*. *lactis* MG1363 was used as input for gene network reconstruction. Prior to this, data exploratory analysis was performed to compare the distributions of the normalized mean and median expressed signals (Fig A, panel A in S1 File) and the density distributions of the Pearson correlation coefficients and Spearman correlation coefficients (Fig A panel B in S1 File). These two plots have similar density distributions except for a shift in the center of the measure of central tendency, namely the mean and median values. Overall, they provide an overview of the distribution of the correlation coefficients and indicate the quantity of high, medium and low correlation values. A summary of parameters resulting from the comparison of the structural properties of the *L*. *lactis* MG1363 co-expression networks to those of the gold-standard networks of *E*. *coli* K-12 [46] and *B*. *subtilis* 168 [47] is shown in Table B in S1 File.

We iteratively evaluated the performance of five network reconstruction methods, namely Pearson correlation, Spearman correlation, GeneNet, SPACE and WGCNA. For computing the adjacency matrix, we used a soft-power threshold value of 5. This value was determined based on the lowest power for which the scale-free model fits the data. Network structural properties such as the number of edges and the module sizes were compared to those of the gold-standard networks. For non-randomly connected biological networks, high modularity is a key indicator of high structural robustness (Fig B in S1 File) [50]. Modular GCNs have hubs in each module, which represent genes for TFs that are crucial for regulation of the genes in the network (Fig B in S1 File). Using the five network reconstruction methods, we searched for modular GCNs with a ratio between the number of edges to the number of genes (***n***_***e***_**/*n***_***g***_) approximating those of the *E*. *coli* K-12 and *B*. *subtilis* 168 networks. Using this ratio criterion, we generated an *L*. *lactis* MG1363 GCN for each of the five methods. The number of lowly connected genes in the *L*. *lactis* MG1363 networks was marginally higher than those in the gold standards. To obtain a high modularity (Q **≈** 0.80) in the networks, a stringent parameter threshold of ***r*** ≈ 0.80 was used for the Pearson correlation or Spearman correlation and the WGCNA. A lower threshold parameter (ρ ≈ 0.70) was required for SPACE (Fig B panel C in S1 File, see also equation A3 in S2 File) and WGCNA (Fig B panel D in S1 File) to prevent a significant reduction of genes and edges in the network, which would result in a very sparse network. More on modularity and community structures in networks can be found in the work of Newman [51]. A further analysis shows that SPACE and WGCNA yield less dense and less modular networks than those generated by Pearson or Spearman correlation (Table C in S1 File). This is deduced from the ratio ***n***_***e***_**/*n***_***g***_ and from the network modularity Ԛ [41,52]. SPACE yielded networks with modules of various sizes and on the lower bound the networks had on average a value of ***n***_***e***_**/*n***_***g***_ of about 6.5 (Table C in S1 File). This is a decent value since many connected genes in a network do not have a regulatory function and most TFs only regulate a few genes [53]. By considering networks corresponding to the plots in Fig B in S1 File and using only networks of approximately the same size (about the same number of genes and edges), the resultant GCNs from using the Pearson correlation coefficients or Spearman correlation coefficients were more densely connected and less modular than those obtained from the other three methods, especially for max(Ԛ) ≈ 0.50 (Fig B in S1 File). Previous studies have shown that using different network reconstruction methods on the same dataset may yield varying network structures [21,22]. In our case, using the Pearson correlation coefficient or Spearman correlation coefficient of 0.90 leads to a near scale-free behavior of the obtained networks (Fig B panel C in S1 File Fig).

**Fig 2:**
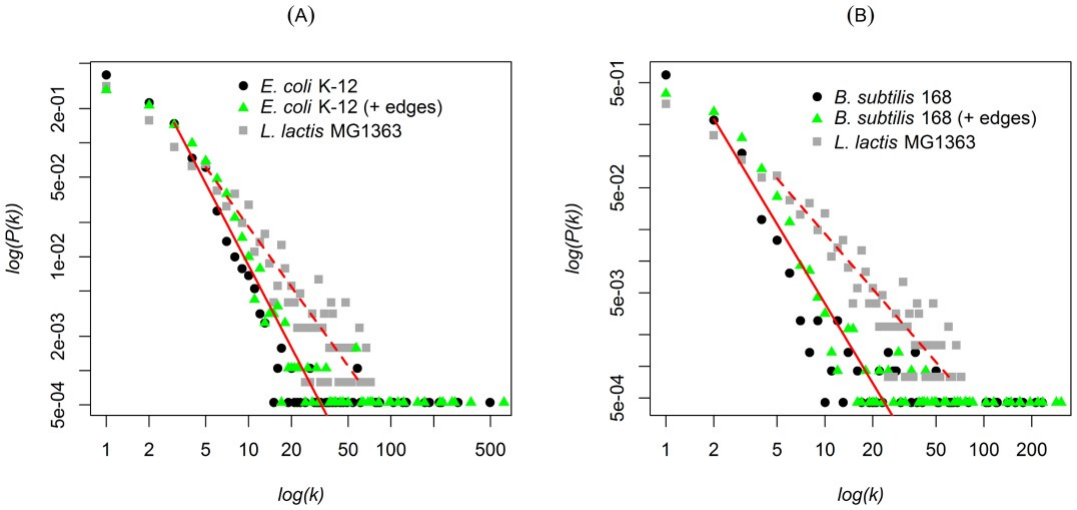
Bench-marking *L*. *lactis* MG1363 SPACE network to gold-standards. A: Degree distribution plot for the *E*. *coli* K-12 network (black circles). *E*. *coli* K-12 (+ edges) represents degree distributions of the network with random edge addition (green triangles). The *x*-axis shows the log-degree distribution (*k*); *y-*axis shows the log-probability of the degree distributions. B: The same as in A for the *B*. *subtilis* 168 and *B*. *subtilis* 168 (+ edges) plots. The criterion for edge addition is described in S2 File. The degree distribution of the *L*. *lactis* MG1363 network is plotted as grey squares in panels A and B. The red dotted lines show the power-law fit to the degree distributions of the *L*. *lactis* MG1363 network.

The structural connectivity of the *L*. *lactis* MG1363 GCN generated using SPACE (Fig 2) was fitted with the power law distribution model (Supplementary Material, S2 File). This model did not support the GCNs obtained using Pearson correlation or Spearman correlation (Figs C and D in S1 File). Using a less stringent threshold parameter results in a large and densely connected network Fig E in S1 File. The term *ρ* in Fig F in S1 File enables pruning of the adjacency matrix to remove spurious weak and non-significant edges between genes [54]. Smaller *ρ* values correspond to increased numbers of enriched gene classes, which is indicated by the total number of significantly enriched Gene Ontology (GO) terms (Fig F panels C and D in S1 File). In these plots, we observed a near-linear relationship with a curve that is similar to that observed between the values of *ρ* and ψ_1_, where ψ_1_ is the average number of GO terms per module with at least one significantly enriched GO term (Supplementary Material, S2 File). Overall, for the network inference, we used the five methods mentioned above to generate networks and subsequently compared and ranked their performances (Table D in S1 File). The results show that SPACE and the WGCNA performed best in the network reconstruction while GeneNet generated the least GO-enriched networks.

### Network module detection

Modules were detected for all networks generated using the five network reconstruction methods. The plots for *ρ* versus ψ_2_ (Fig F panels C and D in S1 File) show that larger ψ_2_ values correspond to the region of the parameter 0.68 ≤ *ρ* ≤ 0.8. Here ψ_2_is the proportion of the total number of significant Fisher’s exact tests (FETs) to the total number of modules with at least one significant GO term—irrespective of the significance of the *p*-value for the FET. The decision of which region in the plots corresponds to a good network is based on how large the ψ_1_ and ψ_2_ values on the vertical axis are and also on the total number of significantly enriched GO terms. This implies that only a specific choice of parameter values results in optimal enrichment of the gene sets in the modules (Fig G in S1 File) of an *L*. *lactis* MG1363 network. Therefore, we selected a range of parameter values and assessed them with respect to ability to yield good quality networks (shaded regions Fig B panels A to E in S1 File; Fig F panels B, D and F in S1 File). Unlike Walk-trap [42], Fast-Greedy [43] and the Infomap community [44] module detection methods, label propagation [45] shows a dip at *ρ* ≈ 0.7 (Fig F panel C and D in S1 File)–which is indicative of a portioned network with only a few lowly enriched modules (low ψ_2_ values) and is attributed to this particular method. Label propagation was relatively slow in partitioning the networks and did not yield modules with the most enriched gene sets. The networks with enriched modules that have the best partitioning were generated using the Walk-trap approach, which was our method of preference after the comparisons. We used it to detect modules in the *L*. *lactis* MG1363 GCNs because it was computationally faster and cheaper and yielded better results (Fig F panels C and D and Fig G in S1 File).

### Structural properties: *L*. *lactis* MG1363 and gold-standard networks

To explore the structural differences between the *L*. *lactis* MG1363 networks and the gold-standard networks, random edges were simulated and added to the *E*. *coli* K-12 and *B*. *subtilis* 168 networks without altering their structural properties (Fig 2). We used the probabilistic random edge addition approach for the edge simulations [55] (S2 File). The *E*. *coli* K-12 and *B*. *subtilis* 168 networks were generated on the basis of literature-validated directed regulatory effects (TFs and their targets). These directed networks were represented as co-expression networks by ignoring the directional regulatory effects and only maintaining edges between genes. The addition of random edges to the gold-standard networks was aimed at explaining any differences in the degree distributions of the *E*. *coli* K-12 and *B*. *subtilis* 168 networks to that of the finally selected *L*. *lactis* MG1363 network, that obtained using SPACE. Fig 2 shows a comparison of the networks of all three organisms. Overall, both the *E*. *coli* K-12 and *B*. *subtilis* 168 networks are less densely connected than that of *L*. *lactis*, even after the addition of random edges (Fig 2A and 2B). The degree distribution plots for the *E*. *coli* K-12 (+ edges) and *B*. *subtilis* 168 (+ edges) networks both shift to the right towards the degree distribution line of the *L*. *lactis* MG1363 network, indicating that differences exist in certain regulatory mechanisms in the organisms. Both the *E*. *coli* and *B*. *subtilis* 168 networks show long-tailed distributions, revealing the presence of TFs such as sigma factors that regulate many targets (typically over 100 genes) [46]. A long tail was absent in the *L*. *lactis* MG1363 network (compare Fig 2A, 2B and Fig B in S1 File); the large sub-networks (regulons) of the pleiotropic regulators CodY and CcpA of *L*. *lactis* [6,56] do reside in the short tail.

**Fig 3:**
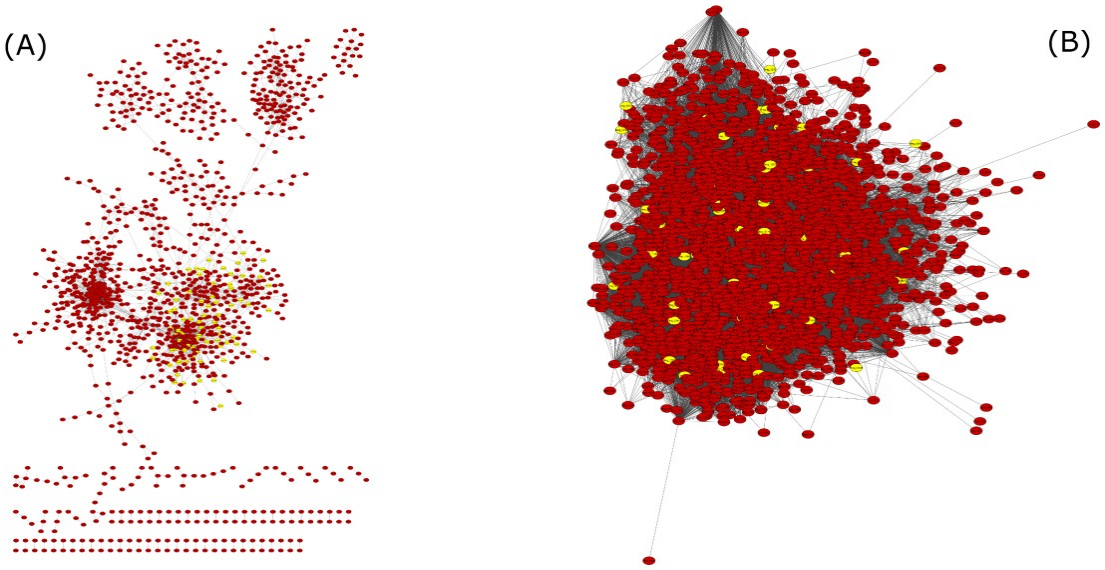
*L*. *lactis* MG1363 GCN visualized in Cytoscape v3.2.0. A: GCN generated using SPACE (ρ = 0.68). Projection of genes (shown in yellow) associated to significantly enriched GO groups in “module 0”, other genes are colored red. The network consists of 1262 genes and 4112 edges. Only genes that satisfied the association threshold levels for inclusion in the adjacency matrix are shown in the network. B: Example network of *L*. *lactis* MG1363 generated using GeneNet (ω = 0.90; 2235 genes and 70386 edges). For instance, the GCN obtained using SPACE has enriched gene sets in “module 0”, which are clustered together in the network (enriched gene sets in yellow), while the same genes are spread out in the GeneNet network.

We chose the network reconstructed using SPACE and *ρ* = 0.68 as the most enriched and informative network for further analysis. Some of the network modules contained hubs, which were defined as genes connected to at least 5 other genes [19,57] (Figs H and I in S1 File). The value *ρ* = 0.68 is stringent but still not all the genes in the regulons that have been studied to date mapped in the GCN (Fig J in S1 File). Genes in the network were assigned to groups of the same ontology (biological processes, cellular components or molecular functions). Our final *L*. *lactis* MG1363 network generated using SPACE comprised of 94 modules, 16 of which contained significantly enriched gene sets (for various GO terms, Table E in S1 File). Only modules that had significantly enriched gene sets these were explored further. The 16 modules contained a varying number of genes with the smallest ones having only two genes and the largest 248 genes. The network is more modular than the one generated using GeneNet (Fig 3A and 3B; see also Fig F panel B in S1 File, which yielded the least modular networks from all the methods used for the network reconstruction).

#### Gene-set enrichment analysis also shows that SPACE generates the best *L*. *lactis* MG1363 network

All five network reconstruction methods were scrutinized for the gene-set enrichment in the network modules they generate (Fig F panels A to F in S1 File) in order to generate the *L*. *lactis* MG1363 GCN of choice. The selection was based on: (i) how closely the resulting network structure matched those of the gold-standards, and (ii) having biologically relevant (enriched) modules. The analyses probe whether modularity and scale-free behavior positively correlate to the biological enrichment of the gene sets in the modules of the GCNs. The number of enriched gene sets was compared for GCNs obtained using thresholds of different correlation parameter values and *ρ*. The results of the GSEA for GO terms on the *L*. *lactis* MG1363 network modules are provided in Table E in S1 File. Module detection in the GCNs obtained using Spearman correlation or WGCNA was performed using the Walk-trap method. Only a few modules were significantly enriched in the Spearman correlation network (low ψ_2_ values in Fig F panels A and B in S1 File compared to those in Fig F panels C to F in S1 File). This indicates a trade-off in the relationship between network connectivity (densely, moderately and lowly connected) and enrichment for about the same number of genes. Densely connected networks further complicate GSEA since the boundaries between modules in such networks are fuzzy and difficult to detect, e.g. the low modularity (low *Q* values) for the densely connected network resulting from GeneNet (Fig B panel E and Fig C panel B in S1 File). This can also be seen for the network in Fig F panel B in S1 File, which was generated using GeneNet. Low values of ψ_1_ and ψ_2_ indicate less enrichment of GO terms in the modules of the networks acquired with WGCNA and Spearman correlation than those obtained using SPACE (Fig F in S1 File). Densely connected GCNs with a low *Q* may have many enriched GO terms; however, the FETs shows that only a specific range of parameter values for the Spearman correlation coefficient *r*_*S*_ and *ρ* yield a good representation of significantly enriched networks (Fig F in S1 File). These results show that SPACE generates the most biologically enriched and structurally best network (Fig F panel A in S1 File).

**Fig 4:**
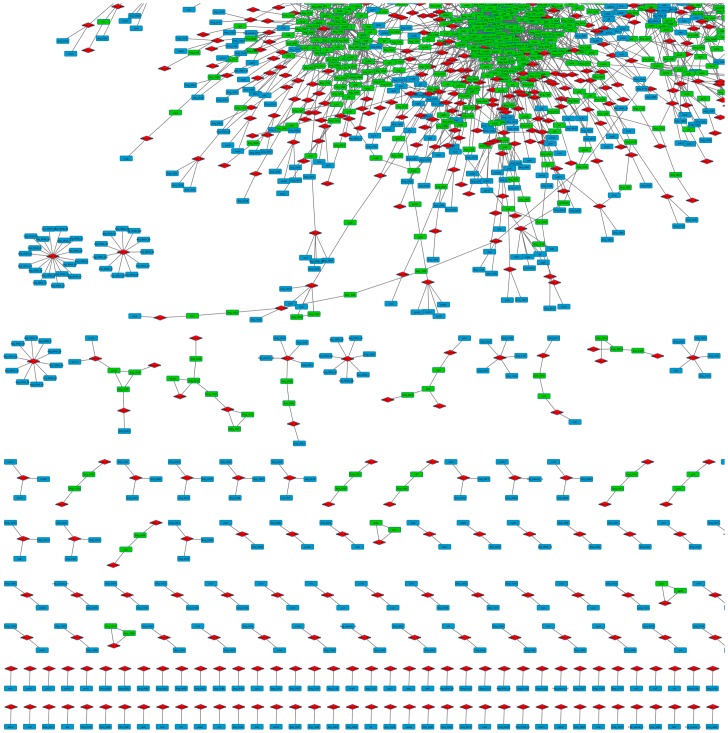
*L*. *lactis* MG1363 GCN integrated with literature-predicted operons (visualized in Cytoscape v3.2.0). The operon IDs are indicated in red, genes predicted to belong to operons are in green, and genes belonging to specific operons based on literature information (http://genome2d.molgenrug.nl) are shown in blue.

We integrated and mapped known operons and regulons from literature onto the *L*. *lactis* MG1363 network reconstructed using SPACE. Thus, genes from 22 regulons were projected on the *L*. *lactis* MG1363 GCN to assess their distribution over the different modules. The results show that genes from the same operon and small regulons (e.g. PurR, HrcA and PyrR) often belong to the same GCN modules (Fig 4). Genes from larger regulons such as CcpA and CodY (Fig K in S1 File) were more broadly distributed over the network. A biological reason might be that genes in the same regulon might not always be co-expressed.

### Enriched network motifs

Nineteen network modules showed evidence of overrepresented motifs (Table G in S1 File). Some genes in a module may be under the control of more than one regulator while a certain regulator may also control the activity of genes in multiple modules (eg, CodY, Fur and LuxR). Additionally, genes can be regulated by multiple other factors, e.g. small RNAs, RNA processing or via co-factor-riboswitch interaction, which could scatter the regulon over multiple modules. Most TFs control the activity of one operon and conserved motifs can only be uncovered by searching the genomes of other organisms for the presence of orthologous DNA patterns. In addition to motifs of the global regulators CodY and CcpA those for more specific regulators such as CtrA, PerR and ArgR were also observed (Table G in S1 File).

### Validation and use of the *L*. *lactis* MG1363 GCN generated using SPACE

To validate the biological relevance of the network modules detected in the *L*. *lactis* MG1363 GCN obtained with SPACE (*ρ* = 0.68), 19 network modules (Tables E and F in S1 File) with at least 5 genes per module were used as input for GSEA. Table 1 contains a summary of these modules and the corresponding overrepresented biological processes within each module. Module 0 and Module 1 are relatively large and predicted to fulfill the general functions transcription regulation and carbohydrate metabolism, respectively. We could associate hypothetical proteins to certain modules and predict their involvement in biological processes. For example, the InterPro IPR017853 protein domain (Glycoside hydrolase, super-family) is represented by 3 genes in Module 1. Two of the genes encode beta-glucosidases while one gene (*llmg_0186*) has no predicted function (Table F in S1 File) but has, apparently, the same expression behavior in many experiments. Indeed, the NCBI link for *llmg_0186* shows that this gene is likely in an operon with the gene for CelB (phosphotransferase system cellobiose-specific component IIC) and is probably involved in sugar (cellobiose) metabolism.

**Table 1:** Enrichment of the most representative biological processes in the modules of the *L*. *lactis* MG1363 GCN.

Module	Members	Over represented function
Module 15	231	Transmembrane transport
Module 0	134	Regulation of transcription
Module 1	93	Carbohydrate metabolic process
Module 9	64	Amino acid transport
Module 2	57	Transmembrane transport
Module 7	25	Phosphoribosyltransferase-like
Module 13	23	General stress proteins
Module 27	14	Acyl-CoA N-acyltransferases
Module 33	11	DNA-binding HTH domain, TetR-type
Module 26	10	Universal stress proteins
Module 25	7	Universal stress proteins

## Conclusions

We have reconstructed and benchmarked the *L*. *lactis* MG1363 GCN using in-house and literature-derived transcriptome data. By analyzing the performance of five network reconstruction methods, namely Pearson correlation, Spearman correlation, WGCNA, GeneNet and SPACE, the latter was shown to yield the best network for *L*. *lactis* MG1363, both by looking at the structure of the network and at the biological content of the modules. The differences in network structure and corresponding parameters are attributed to the methods for computing the network adjacency matrices. Functional analyses demonstrated that the obtained network modules have biological relevance. Examination of the *L*. *lactis* MG1363 GCN shows that some regulons are not members of the same module, an indication that genes in such regulons are regulated by multiple transcription factors also in this organism. A list of differentially expressed genes obtained by DNA microarraying or RNA sequencing, or proteins acquired through proteomics experiments, can be projected on the *L*. *lactis* MG1363 GCN in order to uncover gene/protein function.

## Supporting information

S1 File Fig A. Comparison of density distributions. Fig B. Comparison of network properties for different methods. Fig C. Model fit to network degree distribution of *L*. *lactis* MG1363 and the gold-standards. Fig D. Model fit to GCN degree distribution. Fig E. Correlation coefficients and network size. Fig F. Comparing gene-set enrichment of various *L*. *lactis* MG1363 networks. Fig G. Modules in *L*. *lactis* MG1363 network visualized in Cytoscape v3.2.0. Fig H. Hubs in the *L*. *lactis* MG1363 network. Fig I. Summary statistics of hubs in the *L*. *lactis* MG1363 network. Fig J. Annotated genes in major regulons in *L*. *lactis*. Fig K. Distribution of genes in regulons over the *L*. *lactis* MG1363 network. Table A. Curated data used in the network reconstruction. Table B. Comparison of *L*. *lactis* MG1363 GCN structural properties to gold-standards. Table C. Overview of performance measures of GCN reconstruction approaches. Table D. Ranking of performance of methods used to reconstruct *L*. *lactis* MG1363 GCN. Table E. Gene set enrichment of the *L*. *lactis* MG1363 network modules. Table F. Summary of the top-hit results from the GSEA of two large modules in the *L*. *lactis* MG1363 GCN. Table G. Overrepresented DNA sequence motifs in network modules.
(DOCX)

S2 File Supporting methods and supporting results. (DOCX)
