# Supplementary material for: Reconstruction and inference of the Lactococcus lactis MG1363 gene co-expression network
Source: PLoS One. 2019 May 22;14(5):e0214868. doi: 10.1371/journal.pone.0214868 (PMC6530827; doi:10.1371/journal.pone.0214868)
Supplement: S1 File — Fig A. Comparison of density distributions. Fig B. Comparison of network properties for different methods. Fig C. Model fit to network degree distribution of L. lactis MG1363 and the gold-standards. Fig D. Model fit to GCN degree distribution. Fig E. Correlation coefficients and network size. Fig F. Comparing gene-set enrichment of various L. lactis MG1363 networks. Fig G. Modules in L. lactis MG1363 network visualized in Cytoscape v3.2.0. Fig H. Hubs in the L. lactis MG1363 network. Fig I. Summary statistics of hubs in the L. lactis MG1363 network. Fig J. Annotated genes in major regulons in L. lactis. Fig K. Distribution of genes in regulons over the L. lactis MG1363 network. Table A. Curated data used in the network reconstruction. Table B. Comparison of L. lactis MG1363 GCN structural properties to gold-standards. Table C. Overview of performance measures of GCN reconstruction approaches. Table D. Ranking of performance of methods used to reconstruct L. lactis MG1363 GCN. Table E. Gene set enrichment of the L. lactis MG1363 network modules. Table F. Summary of the top-hit results from the GSEA of two large modules in the L. lactis MG1363 GCN. Table G. Overrepresented DNA sequence motifs in network modules. (DOCX) [file pone.0214868.s001.docx]

# Reconstruction and inference of the *Lactococcus lactis* MG1363 gene co-expression network

Jimmy Omony, Anne de Jong, Jan Kok, Sacha A.F.T. van Hijum

**Supporting information**

## Figures

Fig A. Comparison of density distributions. (A) Density plots of the mean and median genes expression. The plots differ mainly in the peak location and less on the variance. Both distributions are mildly positive skewed. (B) Density distributions of Spearman and Pearson correlation coefficients. Density plots for Pearson correlation coefficients (PCCs, $\boldsymbol{r}_{\boldsymbol{P}}$) and Spearman correlation coefficients (SCCs, $\boldsymbol{r}_{\boldsymbol{S}}$) with: (i) negative values, (ii) mirrored (absolute value of the) negative values, (iii) positive values, and (iv) the combination of both negative and positive values. The density distributions show some variation especially at the values 0.6<$\left| \boldsymbol{r}_{\boldsymbol{P}} \right|$=$\left| \boldsymbol{r}_{\boldsymbol{S}} \right|$≈1 and 0.1<$\left| \boldsymbol{r}_{\boldsymbol{P}} \right|$=$\left| \boldsymbol{r}_{\boldsymbol{S}} \right|$≈0.4. The combined density for the PCCs and SCCs has an inverted and skewed distribution with an off-set at the zero mark ($\left| \boldsymbol{r}_{\boldsymbol{P}} \right|$=$\left| \boldsymbol{r}_{\boldsymbol{S}} \right|$≈0$\boldsymbol{)}$.

Fig B. Comparison of network properties for different methods. The graphs indicate how both (i) number of edges ($\boldsymbol{n}_{\boldsymbol{e}}$) and (ii) modularity vary with changes in the threshold parameters. A and B: The filled green triangle ($\boldsymbol{Q}_{\boldsymbol{E. coli}}$=0.51) and red diamond ($\boldsymbol{Q}_{\boldsymbol{B. subtilis}}$=0.75) are the modularity values for the *E. coli* K-12 and *B. subtilis* 168 networks, respectively. C: SPACE consistently yields modular GCNs. Increasing the threshold parameter generally results in an exponential decrease in $\boldsymbol{n}_{\boldsymbol{e}}$ and increase in modularity (Ԛ). Here ω is the partial correlation coefficient. In C and D, for a wide range of ρ, the Ԛ values for the *L. lactis* MG1363 GCN are comparable to those for the *E. coli* K-12 and *B. subtilis* 168 networks. E: The influence of varying ω on $\boldsymbol{n}_{\boldsymbol{e}}$ and modularity for GCNs generated using GeneNet. Both $\boldsymbol{Q}_{\boldsymbol{E. coli}}$ and $\boldsymbol{Q}_{\boldsymbol{B. subtilis}}$are outside the plotted range of parameters for GeneNet and that for *E. coli* K-12 in SPACE. The blue planes are regions of association parameter that generate the most enriched networks.

Fig C. Model fit to network degree distribution of *L. lactis* MG1363 and the gold-standards. Degree distribution plots for various thresholds in the Pearson correlation coefficient (PCC, $\boldsymbol{r}_{\boldsymbol{P}}$). The red, blue and green lines are the power-law distribution (PLD), truncated power-law distribution (TPLD) and exponential truncated power-law distribution (ETPLD) model fits to data, respectively. The corresponding parameters are given in S2 Table. The plots show that a few nodes are connected with many edges while many nodes are only connected with a small number of edges. Log-log model fit to data for the *E. coli* K-12 GCN (E and F; red line) deviates from the power-law. The deviation is mainly caused by the presence of some nodes with high connectivity ($\boldsymbol{r}_{\boldsymbol{g}}$>50). The TPLD fits the degree distribution of the *L. lactis* MG1363 GCN better than the PLD model (A to D). For the *L. lactis* MG1363 GCNs based on correlation coefficients between 0.70 and 0.90, the power-law (red line) fits the data well, but the network degree distribution pattern changes for increased correlation coefficient (≥0.95). Removing auto-regulatory effects from the *E. coli* K-12 network does not significantly alter its degree distribution (compare E and F). In all the plots (A to F), many nodes have few edges and only a few hubs have a large number of targets (especially for *E. coli* K-12 and *L. lactis* MG1363 GCN with a correlation coefficient of ≤0.9). For $\boldsymbol{k}$>20 we have $\boldsymbol{P}\left( \boldsymbol{k} \right)$≈0 in TPLD (plots E and F). The *L. lactis* MG1363 GCN has a “long-tailed” distribution (A to C) typical of the presence of hubs.

Fig D. Model fit to GCN degree distribution. Degree distributions and model fits to the *L. lactis* MG1363 GCNs generated using Pearson correlation coefficients (PCCs) and Spearman correlation coefficients (SCCs). The truncated power-law distribution (TPLD, blue lines) is a better model fit than the power-law distribution (PLD, red lines). Black circles and green squares are degree distributions for GCNs generated using PCC (S2 Fig) and SCCs, respectively. For stringent thresholds (>0.95), the GCN becomes even scarcer when using SCC compared to using PCCs (plot D). Overall, the degree distributions are similar, especially for larger PCCs and SCCs. Using PCCs yield networks with degree distributions with broad shoulders and larger modules than SCCs.

Fig E. Correlation coefficients and network size. Plots of the relationship between the correlation threshold and the number of edges in the *L. lactis* MG1363 Pearson or Spearman generated networks. The range of correlation coefficient values between 0.8 and 0.9 (indicated by the red dotted lines) was further used as the most realistic for the GCN reconstruction. Using the Spearman correlation coefficients ($\boldsymbol{r}_{\boldsymbol{S}}$) yields less connected networks than with Pearson correlation coefficients ($\boldsymbol{r}_{\boldsymbol{P}}$).

**Fig F. Comparing gene-set enrichment of various *L. lactis* MG1363 networks.** A and B: Influence of varying the correlation parameter ($r_{S}$) on network enrichment for the Spearman correlation coefficient (SCC). The shaded rectangular regions are the most interesting for enriched networks. These figures show the results of a comparative analysis of the performance of four module detection methods with the corresponding GSEA indicator. Overall, the Walk-trap and fast-greedy methods yielded the most enriched network modules. C and D: SPACE: The average number of significant FETs per module ($\Psi_{1}$) differs for different module detection methods although the overall pattern remains similar. There are also variations in $\Psi_{2}$, which represents the proportion of the total number of significant FETs to the total number of modules with at least a single GO group (S1 File). However, the overall patterns of $\Psi_{2}$ vary with module detection methods. The total number of GOs reduces with increasing $\rho$. There is variation in performance between the module detection methods. Both figures show the relationship between the total number of enriched gene sets and the corresponding significance of FET. E and F: WGCNA: Enrichment of the network varies with $\rho$. The regions in gray have the most enriched and structurally sound network with a large number of genes.

Fig G. Modules in *L. lactis* MG1363 network visualized in Cytoscape v3.2.0. Genes belonging to the same module are indicated using the same color. The network generated using SPACE (ρ=0.68) contains 1262 genes and 4112 edges.

**Fig H. Hubs in the *L. lactis* MG1363 network**. In total 471 hubs (yellow) were detected in the *L. lactis* MG1363 network described in S7 Fig. The non-hub genes are shown in red.

**Fig I. Summary statistics of hubs in the *L. lactis* MG1363 network.** (A) Frequency distribution of highly connected nodes was extracted from the network presented in S7 Fig. Hub activity affects many genes in the network and drives specific functions, as seen in *E. coli* [56]*.* (B) Most hubs have less than 25 connections while a few connect to many more genes (up to 72).

**Fig J. Annotated genes in major regulons in *L. lactis*.** Comparison of the number of genes in GCN and genes in the corresponding annotated regulons in *L. lactis*. The mapping is based on GCNs generated using SPACE [22].

Fig K. Distribution of genes in regulons over the *L. lactis* MG1363 network. The network, consisting of 1262 genes and 4112 edges, onto which the CcpA and CodY regulons are colored purple and red, respectively. Localization of genes controlled by CcpA was more conserved than that of genes belonging to the CodY regulon. A number of genes from both regulons cluster in close proximity, with only a few indirect links between genes.

**Fig L. Overrepresented DNA sequence motifs in network modules.**

Output of a DNA sequence motif search (<http://meme-suite.org/>) performed on upstream regions of genes that are part of the indicated network modules. Shown are overrepresented DNA motifs in modules with at least 5 genes that have an upstream intergenic region larger than 20 bases. ^#^, Number of intergenic regions in the module in which the motif is present. Transcription Factor and its cognate *p*-value (in column 5) are derived from a MEME-TomTom search of the DNA motif against the Prokaryote Transcription Factor Bindings Sites database. ^$^, from *Pseudomonas aeruginosa*.

## Tables

**Table A. Curated data used in the network reconstruction.** Summary of data extracted from GEO, NCBI and curated data. Available at URL: <https://figshare.com/s/1cf162124d2cbf25d32f>, DOI: 10.6084/m9.figshare.5450518.

**Table B. Comparison of *L. lactis* MG1363 GCN structural properties to gold-standards**

| Model | $r_{P}$ | $r_{S}$ | $\hat{\beta}\pm\mathrm{sd}\left( \hat{\beta} \right)$ | $\hat{\gamma}\pm\mathrm{sd}\left( \hat{\gamma} \right)$ | $\hat{\alpha}\pm\mathrm{sd}\left( \hat{\alpha} \right)$ | $R^{2}$ | $\hat{C}\left( n_{m} \right)$ | $\left[ n_{g};n_{e} \right]$ | Strain |
| --- | --- | --- | --- | --- | --- | --- | --- | --- | --- |
| PLD | 0.70 | - | 0.040$\pm$0.001 | 0.915$\pm$0.009 | - | 0.838 | 0.321 (20) | [2254;132455] | *L. lactis* MG1363 |
| TPLD | 0.70 | - | 0.0339$\pm$1e-4 | 0.359$\pm$0.017 | 0.012$\pm$1e-4 | 0.882 | “ ” | “ ” | “ ” |
| PLD | - | 0.70 | 0.034$\pm$0.001 | 0.518$\pm$0.009 | - | 0.845 | 0.446 (21) | [2249;98284] | “ ” |
| TPLD | - | 0.70 | 0.029$\pm$0.001 | 0.368$\pm$0.015 | 0.005$\pm$4e-4 | 0.899 | “ ” | “ ” | “ ” |
| PLD | 0.80 | - | 0.114$\pm$0.001 | 0.848$\pm$0.009 | - | 0.965 | 0.285 (234) | [1942;47227] | *L. lactis* MG1363 |
| TPLD | 0.80 | - | 0.111$\pm$0.001 | 0.735$\pm$0.015 | 0.013$\pm$0.002 | 0.969 | “ ” | “ ” | “ ” |
| PLD | - | 0.80 | 0.115$\pm$0.001 | 0.781$\pm$0.009 | - | 0.962 | 0.577 (96) | [1915;26563] | “ ” |
| TPLD | - | 0.80 | 0.109$\pm$0.002 | 0. 644$\pm$0.016 | 0.013$\pm$0.001 | 0.979 | “ ” | “ ” | “ ” |
| PLD | 0.90 | - | 0.213$\pm$0.004 | 1.044$\pm$0.020 | - | 0.975 | 0.549 (123) | [1006;6984] | *L. lactis* MG1363 |
| TPLD | 0.90 | - | 0.213$\pm$0.004 | 1.066$\pm$0.032 | -0.004$\pm$0.004 | 0.976 | “ ” | “ ” | “ ” |
| PLD | - | 0.90 | 0.371$\pm$0.005 | 1.265$\pm$0.023 | - | 0.994 | 0.739 (114) | [787;1816] | “ ” |
| TPLD | - | 0.90 | 0.389$\pm$0.006 | 1.069$\pm$0.038 | 0.063$\pm$0.013 | 0.997 | “ ” | “ ” | “ ” |
| PLD | 0.95 | - | 0.348$\pm$0.016 | 1.096$\pm$0.065 | - | 0.965 | 0.839 (64) | [380,636] | *L. lactis* MG1363 |
| TPLD | 0.95 | - | 0.407$\pm$0.023 | 0.560$\pm$0.139 | 0.194$\pm$0.055 | 0.985 | “ ” | “ ” | “ ” |
| PLD | - | 0.95 | 0.827$\pm$0.003 | 2.692$\pm$0.034 | - | 0.999 | 0.951 (35) | [87;53] | “ ” |
| TPLD | - | 0.95 | NA | NA | NA | NA | “ ” | “ ” | “ ” |
| ETPLD^1^ | - | - | 0.618$\pm$0.013 | 0.070$\pm$0.044 | 0.470$\pm$0.021 | 0.998 | - | [1941;4268]^a^ | *E. coli* K-12 |
| ETPLD^2^ | - | - | 0.409$\pm$0.013 | 1.246$\pm$0.051 | - | 0.958 | - | “ ” | “ ” |
| ETPLD^1^ | - | - | 0.618$\pm$0.013 | 0.069$\pm$0.094 | 0.471$\pm$0.046 | 0.998 | - | [1903;4146]^b^ | “ ” |
| ETPLD^2^ | - | - | 0.002$\pm$0.001 | 0.321$\pm$0.081 | - | 0.296 | - | “ ” | “ ” |
| ETPLD^1^ | - | - | 1.290$\pm$0.152 | 0.239$\pm$0.197 | 0.788$\pm$0.118 | 0.999 | - | [2144;3220]^a^ | *B. subtilis* 168 |
| ETPLD^2^ | - | - | 0.002$\pm$0.001 | 0.305$\pm$0.088 | - | 0.263 | - | “ ” | “ ” |
| ETPLD^1^ | - | - | 1.291$\pm$0.152 | 0.239$\pm$0.197 | 0.788$\pm$0.118 | 0.999 | - | [2103;3143]^b^ | “ ” |
| ETPLD^2^ | - | - | 0.002$\pm$0.001 | 0.308$\pm$0.089 | - | 0.275 | - | “ ” | “ ” |

Parameter estimates for degree distributions of the *L. lactis* MG1363 network reconstructed using Pearson and Spearman correlation coefficients. $C$ is the network clustering coefficient, $n_{m}$ – number of modules. $n_{g}$ and $n_{e}$ - number of genes and edges in a network, respectively; sd - standard deviation. Where $\beta$, $\gamma$, $\alpha$ are model parameters; $R^{2}$ - fraction of variation explained by the model. ^a,b^ Correspond to the *E. coli* K-12 transcription regulatory network (regulonDB) and *B. subtilis* 168 (SubtiWiki database) with and without self-regulation, respectively. $k_{\mathrm{crit}}=13$ was used for both the *E. coli* K-12 and *B. subtilis* 168 networks. ^1,2^ Parameter index of the exponential truncated power-law distribution (ETPLD) model in the first and second components of the model, respectively. (T)PLD – (truncated) power-law distribution. ^NA^ No model fit attained.

**Table C. Overview of performance measures of GCN reconstruction approaches**

| **Method** |  | $Q$ | $D$ | $l_{G}$ | $ND$ | $n_{m}$ | $n_{g}$ | $n_{e}$ | $n_{e}/n_{g}$ |
| --- | --- | --- | --- | --- | --- | --- | --- | --- | --- |
| **PCC** |  |  |  |  |  |  |  |  |  |
| lower $r_{S}$ | 0.70 | 0.3208 | 10 | 3.0929 | 0.0541 | 20 | 2254 | 137455 | 60.98 |
| upper $r_{S}$ | 0.94 | 0.7706 | 14 | 5.2330 | 0.0093 | 77 | 512 | 1212 | 2.38 |
| **SCC** |  |  |  |  |  |  |  |  |  |
| lower $r_{P}$ | 0.70 | 0.4461 | 9 | 3.2532 | 0.0388 | 21 | 2249 | 98284 | 43.70 |
| upper $r_{P}$ | 0.94 | 0.9258 | 8 | 2.8274 | 0.0094 | 57 | 183 | 156 | 0.85 |
| **WGCNA** |  |  |  |  |  |  |  |  |  |
| lower $\rho$ | 0.30 | 0.4054 | 30 | 6.5790 | 0.0221 | 392 | 1546 | 26439 | 17.10 |
| upper $\rho$ | 0.78 | 0.8667 | 6 | 2.3778 | 0.0134 | 41 | 148 | 146 | 0.98 |
| **SPACE** |  |  |  |  |  |  |  |  |  |
| lower $\rho$ | 0.60 | **0.6551** | **15** | **4.8715** | **0.0065** | **58** | **2004** | **13024** | **6.50** |
| upper $\rho$ | 0.84 | **0.8203** | **7** | **2.8179** | **0.0135** | **47** | **184** | **228** | **1.24** |
| **GeneNet** |  |  |  |  |  |  |  |  |  |
| lower $\omega$ | 0.60 | 0.2107 | 10 | 2.1037 | 0.0262 | 4 | 2302 | 138880 | 60.33 |
| upper $\omega$ | 0.96 | 0.2854 | 12 | 2.5229 | 0.0104 | 5 | 2173 | 49125 | 22.61 |

The parameters $r_{S}$, $r_{P}$, ρ and ω are based on the threshold values for making the adjacency matrix (Methods). The Pearson correlation coefficient (PCC) and Spearman correlation coefficient (SCC), WGCNA and SPACE had vastly different network structural properties from those of GeneNet. GeneNet yielded densely connected GCNs with low modularity. There was also some variation in the structural properties of the GCNs generated using the first four methods above. Such variation might have a significant impact on the enrichment of the gene classes in the network modules. Network properties corresponding to the lower and upper parameter thresholds for the various methods are presented in this Table for the different reconstruction methods. The other network structural properties are shown in S2 Fig, $D$ – GCN diameter and $l_{G}$ – mean path length of network; $ND$ refers to the GCN density; $n_{m}$ is the number of modules; $n_{g}$ and $n_{e}$ are the number of genes and edges, respectively.

Table D. Ranking of performance of methods used to reconstruct *L. lactis* MG1363 GCN. This performance ranking is based on comparisons of network structural properties and gene set enrichment in the network modules.

| **Method** | **Similarity measure** | **Preference** |
| --- | --- | --- |
| SPACE | Partial correlations [22] | First |
| WGCNA | Partial correlations [23] and this work | Second^a^ |
| Pearson and Spearman correlation | Correlation coefficients [57,58] | Second^b^ |
| GeneNet | Partial correlations [21] | Third |

^a,b^ The ranking depends on the influence of the threshold parameters on the network structural properties (S4 Fig) and biological enrichment.

**Table E. Gene set enrichment of the *L. lactis* MG1363 network modules**

| **CLASS** | **Top-Hits**  **(Class size)** | **Adj-**$\boldsymbol{p}$**-values** | **Description** |
| --- | --- | --- | --- |
| **Module 0** |  |  |  |
| GO:0006412 | 23(52) | 1.78E-10 | translation |
| GO:0005622 | 26(70) | 2.29E-10 | intracellular |
| GO:0003735 | 22(50) | 2.29E-10 | structural constituent of ribosome |
| GO:0005840 | 21(47) | 3.95E-10 | ribosome |
| GO:0003824 | 25(123) | 3.43E-4 | catalytic activity |
| GO:0003899 | 4(4) | 7.35E-4 | DNA-directed RNA polymerase activity |
| **Module 1** |  |  |  |
| GO:0008804 | 2(2) | 5.91E-3 | carbamate kinase activity |
| GO:0015604 | 2(2) | 5.91E-3 | organic phosphonate transmembrane transporter activity |
| GO:0016743 | 2(2) | 5.91E-3 | carboxyl- or carbamoyltransferase activity |
| GO:0005887 | 2(3) | 7.48E-3 | integral component of plasma membrane |
| GO:0006012 | 2(3) | 7.48E-3 | galactose metabolic process |
| GO:0015716 | 2(3) | 7.48E-3 | organic phosphonate transport |
| **Module 2** |  |  |  |
| GO:0006355 | 2(45) | 0.01865 | regulation of transcription, DNA-templated |
| GO:0016021 | 2(61) | 0.01865 | integral component of membrane |
| GO:0000166 | 2(84) | 0.02326 | nucleotide binding |
| **Module 3** |  |  |  |
| GO:0016491 | 12(52) | 2.77E-5 | oxidoreductase activity |
| GO:0005975 | 8(34) | 1.22E-3 | carbohydrate metabolic process |
| GO:0016021 | 9(61) | 1.21E-2 | integral component of membrane |
| GO:0055114 | 9(64) | 1.31E-2 | oxidation-reduction process |
| GO:0055085 | 5(24) | 2.44E-2 | transmembrane transport |
| GO:0016020 | 10(88) | 2.44E-2 | membrane |
| **Module 4** |  |  |  |
| GO:0016020 | 3(88) | 0 | membrane |
| GO:0005524 | 2(116) | 0.01578 | ATP binding |
| **Module 5** |  |  |  |
| GO:0005524 | 3(116) | 0 | ATP binding |
| GO:0003824 | 2(123) | 0.01775 | catalytic activity |
| **Module 6** |  |  |  |
| GO:0003824 | 3(123) | 7.01E-3 | catalytic activity |
| GO:0000166 | 2(84) | 0.03489 | nucleotide binding |
| GO:0003677 | 2(116) | 0.04342 | DNA binding |
| **Module 7** |  |  |  |
| GO:0016787 | 6(50) | 7.98E-3 | hydrolase activity |
| GO:0003333 | 3(9) | 7.98E-3 | amino acid transmembrane transport |
| GO:0015171 | 3(9) | 7.98E-3 | amino acid transmembrane transport |
| GO:0016620 | 2(3) | 0.01017 | oxidoreductase activity |
| GO:0009401 | 2(9) | 0.07520 | phosphoenolpyruvate-dependent PTS |
| GO:0055085 | 3(24) | 0.07520 | transmembrane transport |
| **Module 8** |  |  |  |
| GO:0005515 | 3(20) | 1.01E-3 | protein binding |
| GO:0005524 | 3(116) | 0.08805 | ATP binding |
| GO:0008152 | 2(69) | 0.13661 | metabolic process |
| GO:0016020 | 2(88) | 0.15789 | membrane |
| GO:0003677 | 2(116) | 0.18549 | DNA binding |
| GO:0003824 | 2(123) | 0.18549 | catalytic activity |
| **Module 9** |  |  |  |
| GO:0005840 | 2(47) | 9.07E-3 | ribosome |
| GO:0003735 | 2(50) | 9.07E-3 | structural constituent of ribosome |
| GO:0006412 | 2(52) | 9.07E-3 | translation |
| **Module 11** |  |  |  |
| GO:0000166 | 3(84) | 6.31E-3 | nucleotide binding |
| GO:0016020 | 3(88) | 6.31E-3 | membrane |
| GO:0008152 | 2(69) | 0.04019 | metabolic process |
| GO:0003824 | 2(123) | 0.08869 | catalytic activity |
| **Module 15** |  |  |  |
| GO:0003824 | 3(123) | 0.03376 | catalytic activity |
| GO:0016491 | 2(52) | 0.03496 | oxidoreductase activity |
| GO:0016020 | 2(88) | 0.06371 | membrane |
| GO:0005524 | 2(116) | 0.07972 | ATP binding |
| **Module 16** |  |  |  |
| GO:0009396 | 2(3) | 6.38E-5 | folic biosynthetic process |
| GO:0055114 | 2(64) | 2.05E-2 | oxidation-reduction process |
| GO:0003824 | 2(123) | 4.86E-2 | catalytic activity |
| **Module 18** |  |  |  |
| GO:0008152 | 3(69) | 0 | metabolic process |
| **Module 34** |  |  |  |
| GO:0005840 | 2(47) | 0.02331 | ribosome |
| GO:0003735 | 2(50) | 0.02331 | structural constituent of ribosome |
| GO:0006412 | 2(52) | 0.02331 | translation |
| GO:0005622 | 2(70) | 0.03098 | intracellular |
| **Module 37** |  |  |  |
| GO:0006259 | 2(2) | 3.48E-4 | DNA metabolic process |
| GO:0003916 | 2(3) | 5.19E-4 | DNA topoisomerase activity |
| GO:0003918 | 2(4) | 6.90E-4 | DNA topoisomerase type II activity |
| GO:0006265 | 2(5) | 8.60E-4 | DNA topological change |
| GO:0005694 | 2(6) | 1.03E-3 | chromosome |
| GO:0003677 | 2(116) | 0.21727 | DNA binding |

Results of the Hypergeometric distribution test with the GO classes and the corresponding gene set enrichment analysis outputs for the *L. lactis* MG1363 GCN. A total of 16 of the 94 modules were significantly enriched for biological processes. Most small-sized modules (with less than 5 genes) contained no enriched gene classes.

**Table F. Summary of the top-hit results from the GSEA of two large modules in the *L. lactis* MG1363 GCN**

| **Module** | **CLASS ID** | **Top-Hits**  **(Class size)** | **Adj.**  $\boldsymbol{p}$**-values** | **Description** |
| --- | --- | --- | --- | --- |
| **Module 0** | GO:0003677 | 18(221) | 5.00E-09 | DNA binding |
|  | GO:0005975 | 11(76) | 1.10E-07 | carbohydrate metabolic process |
|  | GO:0008152 | 12(140) | 5.60E-06 | metabolic process |
|  | GO:0043565 | 7(41) | 1.60E-05 | sequence-specific DNA binding |
|  | GO:0003700 | 8(65) | 2.90E-05 | sequence-specific DNA binding transcription factor activity |
|  | GO:0006355 | 9(94) | 4.60E-05 | regulation of transcription, DNA-templated |
|  | GO:0004553 | 5(21) | 5.80E-05 | hydrolase activity, hydrolyzing O-glycosyl compounds |
|  | GO:0016491 | 8(87) | 1.70E-04 | oxidoreductase activity |
|  | GO:0003824 | 11(190) | 3.20E-04 | catalytic activity |
|  | GO:0046685 | 2(3) | 2.10E-03 | response to arsenic-containing substance |
| **Module 1** | GO:0005975 | 10(76) | 3.50E-06 | carbohydrate metabolic process |
|  | GO:0003824 | 9(190) | 1.50E-02 | catalytic activity |
|  | GO:0004553 | 3(21) | 1.50E-02 | hydrolase activity, hydrolyzing O-glycosyl compounds |

**Table G Overrepresented DNA sequence motifs in network modules**

| **Module** | **Logo** | **Number^#^** | **Transcription Factor** | ***p*-value** |
| --- | --- | --- | --- | --- |
| Module 0 | 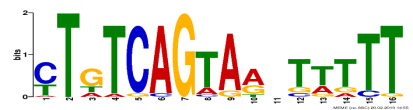 | 19 | CodY | 1.45e-03 |
| Module 1 | 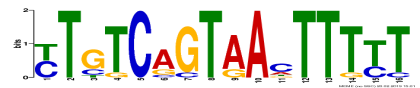 | 10 | CodY | 1.61e-03 |
| Module 2 | 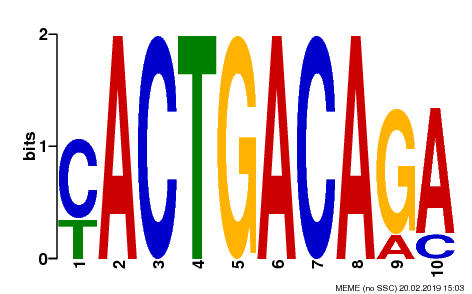 | 6 | OxyR | 1.29e-02 |
| Module 3 | 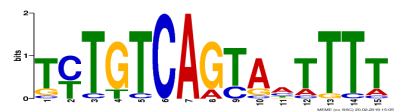 | 12 | CodY | 1.32e-03 |
| Module 6 | 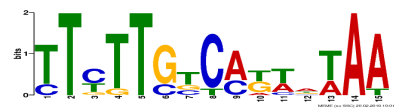 | 9 | Fur | 1.72e-02 |
| Module 7 | 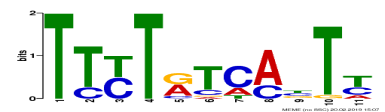 | 18 | CsgD | 2.07e-03 |
| Module 7 | 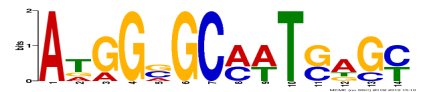 | 5 | ArgR | 4.10e-03 |
| Module 8 | 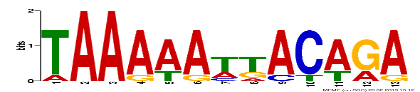 | 8 | PerR | 9.11e-03 |
| Module 9 | 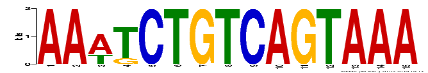 | 6 | LuxR | 4.32e-03 |
| Module 10 | 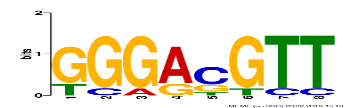 | 8 | GlnR | 2.72e-03 |
| Module 11 | 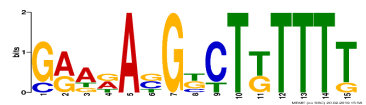 | 5 | CcpA | 1.81e-04 |
| Module 13 | 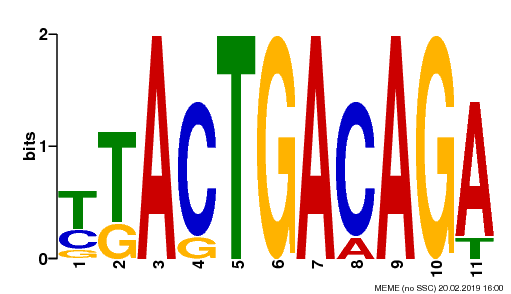 | 7 | LuxR | 3.92e-02 |
| Module 15 | 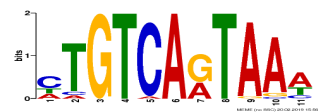 | 30 | CodY | 2.79e-03 |
| Module 18 | 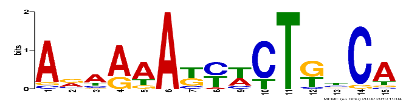 | 14 | Fur | 1.67e-03 |
| Module 25 | 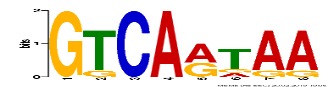 | 7 | LuxR | 2.67e-03 |
| Module 26 | 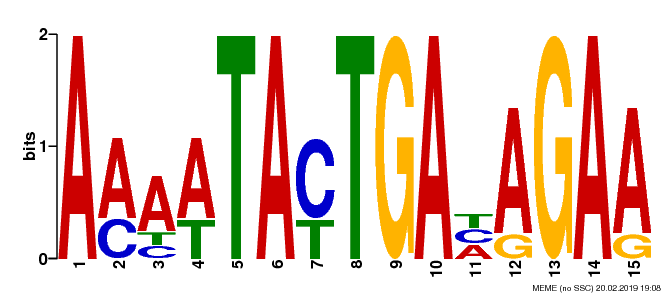 | 6 | CodY | 1.11e-02 |
| Module 27 | 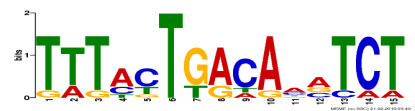 | 8 | CodY | 9.78e-04 |
| Module 32 | 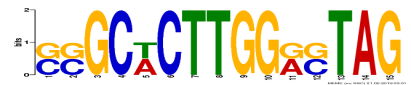 | 15 | VqsM^$^ | 3.35e-03 |
| Module 33 | 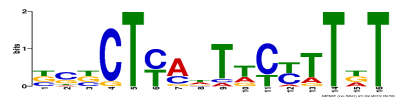 | 11 | CtrA | 1.25e-02 |

## Additional files

**S2 File. Supporting Methods and Supporting Results.**
