# Supplementary material for: Reconstruction and inference of the Lactococcus lactis MG1363 gene co-expression network
Source: PLoS One. 2019 May 22;14(5):e0214868. doi: 10.1371/journal.pone.0214868 (PMC6530827; doi:10.1371/journal.pone.0214868)
Supplement: S2 File — (DOCX) [file pone.0214868.s002.docx]

# Supporting Information

## Reconstruction and Inference of the *Lactococcus lactis* MG1363 Gene Co-expression Network

Jimmy Omony^1,2^, Anne de Jong^1,2^, Jan Kok^1,2,*^, Sacha A.F.T. van Hijum^2,3,4^

^1^Laboratory of Molecular Genetics, University of Groningen, 9747 AG Groningen, The Netherlands.

^2^Top Institute Food and Nutrition (TIFN), Nieuwe Kanaal 9A, 6709 PA Wageningen, The Netherlands.

^3^NIZO Food Research B.V., P.O. Box 20, Ede 6710 BA, Ede, The Netherlands.

*Correspondence: E**-**mail: [jan.kok@rug.nl](mailto:jan.kok@rug.nl)

## Methods

### Data source and diagnostics

The density distribution of the median and mean gene expression values were plotted (Fig A panel A in S1 file). The density plot of the median values was divided into three roughly equal parts by categorizing the genes into groups with: genes with High (H), Medium (M) and Low (L) expression. The preferential inclusion of genes in the gene co-expression network (GCN) was checked after reconstruction with the Pearson and Spearman correlation coefficients. All the three groups were well-represented in the network (in the order M$\gg$L>H groups), irrespective of the Pearson or Spearman correlation coefficient threshold. Using $r=0.90$ results in a network with group sectors of M$\approx$60% and H$\approx$6%. The genes in group H, M and L were combined pair-wise and density plots were made for the median expression values. The density distribution of the combined classes was similar; hence, no preferential representation of genes from the different groups. Overall, the corresponding density plots had similar distributions (Fig A panel B in S1 file); therefore, using the Pearson or Spearman correlation coefficient should yield co-expression networks with similar structural properties.

### GeneNet

GeneNet is based on Graphical Gaussian Models in which partial correlation coefficients are estimated using dynamic shrinkage [1,2]. GeneNet requires less tuning of parameters especially for the probability that an edge exists between nodes. Setting a high partial correlation coefficient threshold ($\omega\geq0.80$) enables the removal of spurious edges from the adjacency matrix. This stringent criterion ensures a low false discovery rate ($<$0.20) [3]. Overall, GeneNet generated the least modular networks (Figs 2E and 3B).

### Power-law distribution (PLD)

It might be unrealistic to consider most nodes as highly or sparsely inter-connected since module size, module composition and network topology vary with the measure of association cut-off. We tested $0.8\leq r\leq0.95$ for the Pearson correlation coefficient and Spearman correlation coefficient based networks. For $n_{g}>1000$ (Fig E in S1 file), $n_{e}$ increases exponentially while $r\geq0.97$ results in about the same number of genes and edges (for $n_{g}\approx n_{e}<100$). This leaves about $5\%$ of the initial 2310 genes in the association network. A fully connected network with $n_{g}$ genes has $n_{e,max}=n_{g}\left( n_{g}-1 \right)/2$ edges and network density of $ND={n_{e}}/{n_{e,max}}$. The *L. lactis* MG1363 GCN from $r\geq0.97$ had $ND*100\approx1.87e-3\ll1\%$. Generally, networks are sparse if $ND*100\ll1\%$.

### Truncated power-law distribution (TPLD)

The TPLD is commonly used to describe the degree distribution of biological networks [4,5] and can be considered as a PLD with a sharp drop-off towards higher degree nodes. It has the form:

$P\left( k \right)=\beta k^{-\gamma}exp\left( -\alpha k \right)$ (A1)

$\beta$ and $\alpha$ are constants and $\gamma$ is the power law exponent, $k_{i}=\sum_{j}a_{ij}$ is the total number of edges from node $i$. Then the probability that node $i$ has $k_{i}$ edges is $P\left( k \right):=P(k_{i})=k_{i}/\sum_{j} k_{j}$ (which is the proportion of nodes with degree $k$).

### Exponential truncated power-law distribution (ETPLD)

The model is:

$P\left( k \right)=\left\{ \begin{matrix} \beta_{1}k^{-\gamma_{1}}\exp\left( -\alpha k \right), \mathrm{for} k<k_{\mathrm{crit}} \\ \beta_{2}k^{-\gamma_{2}}, \mathrm{for} k>k_{\mathrm{crit}} \end{matrix} \right.$ (A2)

$k_{\mathrm{crit}}:=1/\alpha$ is the critical network degree, $\gamma_{1}$ and $\gamma_{2}$ are scale property indicators.

### Network structural properties

To assess the *L. lactis* MG1363 network quality, we used the following measures:

1. **Diameter:** $D_{ij}=max\{d_{ij}|i,j\in N$}, $N$ represents all nodes in a network. The diameter measures the largest distance between any two nodes in a network.
2. **Clustering coefficient (transitivity):** given by $C_{i}=2a_{i}/k_{i}(k_{i}-1)$, where $a_{ij}$ is an edge between nodes $i$ and $j$; $k$ is the node degree (connectivity) [6-8].
3. **Power-law distribution** [9,10]: In co-expression networks, the average clustering coefficient ($C$) is a measure of the number of triangles in a network [11]. The local clustering coefficient for node $i$ is the number of triangles to which it is connected divided by the number of triples around it. The clustering coefficient for the global network is the average of local $C_{i}$ values; $C=\left( 1/{n_{g}} \right)\sum_{i=1}^{n_{g}} C_{i}$; $0\leq C_{i},C\leq1$ and $C_{i}=2a_{i}/k_{i}(k_{i}-1)$. Large $C$ values are associated with high robustness. Using $r<0.90$ leads to densely connected networks (where the number of edges far exceeds the number of genes, $n_{e}\gg n_{g}$) which complicate the module detection.

### Bench-marking network reconstruction methods

The existence of an edge between two nodes in a network is based on a high correlation ($r_{ij}$) in expression values of genes $i$ and $j$. We define the adjacency matrix as $\left[ \rho\right]_{ij}=r_{ij}^{\theta}$; for the Pearson or Spearman correlation coefficients, we have $\theta=1$ while in the WGCNA, an optimal value $\theta=\theta^{*}$ for thresholding the adjacency matrix has to be determined. To investigate the network topology a defined range for $\rho_{ij}$ was specified. The network adjacency matrix is given by the expression:

$\left[ \rho\right]_{ij}=\left\{ \begin{matrix} \left[ \rho_{ij} \right]_{n_{g}\times n_{g}}, \mathrm{if}\rho_{ij}\geq\rho_{ij}^{*} \\ 0, \mathrm{elsewhere} \end{matrix} \right.$ (A3)

For hard thresholding ($\rho_{ij}\geq\rho_{ij}^{*}$), set $\rho_{ij}=1$. Meanwhile, soft thresholding ($0<\rho_{ij}^{*}\leq\rho_{ij}<1$) is preferred for large networks [12]. We pruned the weighted co-expression network to create the corresponding adjacency matrix. In GeneNet, the threshold parameter corresponds to the limiting partial correlation. To evaluate the network connectivity, we define the network density as $D:=\psi\left( \phi\right)$ where $\phi:=r_{P},r_{S},\rho, \omega$; thus, $\phi^{*}\approx min\left( \psi^{-1}\left( ND \right) \right)$ is the optimal threshold value for edge-determination in the adjacency matrix.

### Adding random edges to the gold-standard networks

We used the probabilistic random edge addition (PREA) to assess whether adding random edges to the gold-standard networks account for their structural differences with the *L. lactis* MG1363 GCN. Edges were independently added to the gold-standard networks using the PREA, which involves: determination of the probability of degree connectivity for all nodes in the network. These probabilities were used to generate extra edges in the networks. Bootstrap sampling with replacement was used to determine the source and target nodes for new edge assignment. Redundancies were removed to ensure uniqueness of edges in the new (resultant) network. Genes were considered to have varying regulation potential and a different number of targets. The edge addition criterion is similar to preferential edge attachment model in which nodes rich in connectivity become richer resulting in a scale-free behavior [10]. Therefore, compared to randomly selected nodes, intra-modular hubs have a higher likelihood of being connected to other nodes. The probability of connecting a particular node to any other randomly selected node in a network is:

$P\left( g_{i} \right)={k_{g_{j}}}/{\sum_{j=1}^{n_{g}} k_{g_{j}}}$ where $\sum_{i=1}^{n_{g}} P\left( g_{i} \right)=1$ (A4)

The results for the PREA analysis are given in Fig 2A and 2B.

### Gene Set Enrichment Analysis (GSEA)

We define the first indicator for the performance of the GSEA using the expression:

$\Psi_{1}\left( \lambda_{1},\lambda_{2} \right)={\lambda_{1}}/{\lambda_{2}}$ (A5)

where $\Psi_{1}$ is the average number of GO terms per module with at least one significantly enriched GO term; $\lambda_{1}$ – total number of significant GO terms in all modules and $\lambda_{2}$ – the number of modules with at least one significant GO term. The second indicator is:

$\Psi_{2}\left( \xi_{1},\xi_{2} \right)={\xi_{1}}/\left( \xi_{1}+\xi_{2} \right)$ (A6)

Here $\Psi_{2}$ is the proportion of the total number of significant Fisher’s exact tests (FETs) to the total number of modules with at least one significant GO term – irrespective of the significance of the $p$-value for the FET (S6 Fig). Here $\xi_{1}$ is the total number of significant FETs per module and $\xi_{2}$ is the total number of modules with at least one significant GO term.

## Results

### Degree distribution model fits to network from Pearson and Spearman correlation

For scale-free networks, the parameter $\gamma$ has been estimated to be in the range $2<\gamma<3$ [9,13] although the boundaries of this range remain debated [14,15]. The gold-standard *E. coli* K-12 network [16] elucidates on realistic $\gamma$ values. The parameter estimates for the *L. lactis* MG1363 GCN (Table C in S1 file) show that $\hat{\gamma}$ falls outside $2<\gamma<3$. The well-curated *E. coli* K-12 networks based on experimental data are scale-free (Figs 2A and 2B; and 3E and S3F Figs), but with noticeable features like the large *E. coli* K-12 hubs (sigma-factors) with a high number of connections. Hence, the exponential truncated power-law distribution (ETPLD) is a better fit compared to the PLD and the TPLD models (S3 Fig).

### Network reconstruction using SPACE: degree distribution parameters

Fitting equation (1) to the degree distributions in Fig 2 results in the following parameter estimates for: (i) *E. coli* K-12 GCN: $\hat{\beta}=2.076\pm0.0236$ ($p=$2.84e-9) and $\hat{\gamma}=2.395\pm0.091$ ($p=$2.7e-20); (ii) *B. subtilis* 168 GCN: $\hat{\beta}=1.284\pm0.101$ ($p=$3.92e-16) and $\hat{\gamma}=2.513\pm0.099$ ($p=$1.3e-27); and (iii) *L. lactis* MG1363 GCN: $\hat{\beta}=1.023\pm0.173$ ($p=$ 4.75e-7) and $\hat{\gamma}=1.748\pm0.087$ ($p=$ 8.9e-24).

References

1. Schafer J, Strimmer K. A shrinkage approach to large-scale covariance matrix estimation and implications for functional genomics. Stat Appl Genet Mol Biol. 2005;4: Article32.

2. Opgen-Rhein R, Strimmer K. Inferring gene dependency networks from genomic longitudinal data: a functional data approach. REVSTAT. 2006;4: 53-65.

3. Benjamini Y, Hochberg Y. Controlling the false discovery rate: a practical and powerful approach to multiple testing. J R Statist Soc B. 1995;57: 289-300.

4. Albert R, Jeong H, Barabasi AL. Error and attack tolerance of complex networks. Nature. 2000;406: 378-382.

5. Uetz P, Giot L, Cagney G, Mansfield TA, Judson RS, Knight JR, et al. A comprehensive analysis of protein-protein interactions in Saccharomyces cerevisiae. Nature. 2000;403: 623-627.

6. Guelzim N, Bottani S, Bourgine P, Kepes F. Topological and causal structure of the yeast transcriptional regulatory network. Nat Genet. 2002;31: 60-63.

7. Ma HW, Zeng AP. The connectivity structure, giant strong component and centrality of metabolic networks. Bioinformatics. 2003;19: 1423-1430.

8. Bergmann S, Ihmels J, Barkai N. Similarities and differences in genome-wide expression data of six organisms. PLoS Biol. 2004;2: E9.

9. Khanin R, Wit E. How scale-free are biological networks. J Comput Biol. 2006;13: 810-818.

10. Barabasi AL, Albert R. Emergence of scaling in random networks. Science. 1999;286: 509-512.

11. Watts DJ, Strogatz SH. Collective dynamics of 'small-world' networks. Nature. 1998;393: 440-442.

12. Langfelder P, Horvath S. WGCNA: an R package for weighted correlation network analysis. BMC Bioinformatics. 2008;9: 559.

13. Albert R, Barabási A. Statistical mechanics of complex networks. Rev Modern Phys. 2002;74: 47-97.

14. Zhu X, Gerstein M, Snyder M. Getting connected: analysis and principles of biological networks. Genes Dev. 2007;21: 1010-1024.

15. Choromański K, Matuszak M, MiȩKisz J. Scale-Free Graph with Preferential Attachment and Evolving Internal Vertex Structure. Journal of Statistical Physics. 2013;151: 1175.

16. Salgado H, Peralta-Gil M, Gama-Castro S, Santos-Zavaleta A, Muniz-Rascado L, Garcia-Sotelo JS, et al. RegulonDB v8.0: omics data sets, evolutionary conservation, regulatory phrases, cross-validated gold standards and more. Nucleic Acids Res. 2013;41: D203-13.
